# Empowering healthcare professionals with no-code artificial intelligence platforms for model development, a practical demonstration for pathology

**DOI:** 10.15190/d.2024.1

**Published:** 2024-03-30

**Authors:** Sayed Shahabuddin Hoseini, Rajan Dewar

**Affiliations:** ^1^Department of Pathology, Westchester Medical Center, 100 Woods Rd, Valhalla, NY 10595, USA

**Keywords:** Machine Learning, Teachable Machine, No-Code platforms, Peripheral Blood White Cell Differentials, Hematology

## Abstract

Artificial intelligence (AI) and machine learning based applications are thought to impact the practice of healthcare by transforming diagnostic patient management approaches. However, domain knowledge, clinical and coding expertise are likely the biggest challenge and a substantial barrier in developing practical AI models. Most informatics and AI experts are not familiar with the nuances in medicine, and most doctors are not efficient coders. To address this barrier, a few “no-code” AI platforms are emerging. They enable medical professionals to create AI models without coding skills. This study examines Teachable Machine™, a no-code AI platform, to classify white blood cells into the five common WBC types. Training data from publicly available datasets were used, and model accuracy was improved by fine-tuning hyperparameters. Sensitivity, precision, and F1 score calculations evaluated model performance, and independent datasets were employed for testing. Several factors that influence the performance of the model were tested. The model achieved 97% accuracy in classifying white blood cells, with high sensitivity and precision. Independent validation supported its potential for further development. This is the first study that demonstrates the value of a free no-code algorithm based AI platforms use in hematopathology using authentic datasets for training. It opens an opportunity for the healthcare professionals to get hands-on experience with AI and to create practical AI models without coding expertise.

## INTRODUCTION

In recent years, the field of healthcare has witnessed the emergence of powerful tools such as artificial intelligence (AI) and machine learning^1^. AI has found applications across various facets of the medical field. It can transcribe medical notes, produce patient information summaries, and provide valuable support to insurance companies and payors in the claims process. Substantial efforts have been directed towards integrating AI into the interpretation of medical images, spanning domains like radiology, pathology, optometry, and endoscopy. Furthermore, AI-driven tools are increasingly being employed for the examination and comprehension of extensive research databases, encompassing a wide array of data, from laboratory results to clinical information^2-5^. However, developing AI models in these domains often requires a blend of expertise in both computer science and medical science. The bottleneck/barrier is two ways: computer scientists are not familiar with the nuances of medicine and most physicians and healthcare professionals do not have a significant background in computer science^6^. The new generation of AI platforms is called the no-code platforms. They feature an intuitive graphical interface with simple drag-and-drop functionalities that empowers beginners to create AI models. A significant portion of these platforms operates in the cloud, eliminating the need for installation, maintenance, and related administrative challenges^7^. By leveraging these no-code AI systems, medical professionals can more easily transform their ideas into tangible AI models, empowering them to contribute to the advancements in AI applications within their fields of expertise.

Nevertheless, no-code AI platforms have received limited attention from scholars, despite predictions of their rapid adoption through 2023^7^. One contributing factor is the limited introduction of these platforms to healthcare professionals. We believe that a broader dissemination of practical examples showcasing the use of no-code AI platforms in medicine, along with educational papers and workshops targeting the healthcare professional community, will encourage widespread engagement in such projects by healthcare professionals.

In this study, we set out to examine the capabilities of Teachable Machine, a freely available no-code AI platform, in classifying peripheral blood cells. Our focus was on categorizing white blood cells into five of the most common distinct peripheral blood cell types: neutrophils, lymphocytes, monocytes, eosinophils, and basophils. Through this demonstrative study, we propose that a no-code AI platform can create potential AI models for medical and pathological applications. These tools may help transcend the computing-healthcare boundaries and harness the power of AI to improve patient care.

## MATERIAL AND METHODS

### Data collection

Training cells were obtained from publicly available published datasets^8-10^. These datasets consisted of images captured using CellaVision machine. No images were removed from these databases, and the data were used as published without modification or image enhancements.

### Model development with Teachable Machine

Teachable Machine is a free online platform by Google for machine learning projects without a need for coding experience. It employs TensorFlow.js, a JavaScript machine learning library, to enable users to train and execute their models directly within their web browsers. It provides flexibility in adjusting three key hyperparameters: Epoch, Batch size, and learning rate. In this study, these parameters were fine-tuned to enhance the accuracy of the model. Care was taken to avoid overtraining the model. By default, the training dataset in Teachable Machine consisted of 85% of cells from each class for training purposes, while the remaining 15% were reserved for testing and evaluation.

To independently assess the performance of the developed model, additional testing was conducted using white blood cell images obtained from different sources^9-11^. The sensitivity (recall), precision (positive predictive value), and F1 score were calculated to evaluate the model's performance using the following formulae:

Sensitivity (recall) = True positives / (True positives + False negatives);

Precision = True positives / (True positives + False positives);

F1 score = 2 × (precision × recall) / (precision + recall).

Overall accuracy = the sum of correctly classified cells / total number of cells. The first model is available at https://teachablemachine.withgoogle.com/models/K pPUw24d1/ and the enhanced second model is available at https://teachablemachine.withgoogle.com/models/Vc 8hNMdN1/

A step-by-step visual explanation of how to make an image classification project on Teachable Machine is shown in supplementary material 1.

### Ethical considerations

We used only publicly available datasets with no identifiable patient information or protected healthcare information (PHI) included. Thus, the study qualifies for exemption from institutional IRB requirements.

## RESULTS

**Figure 1:**
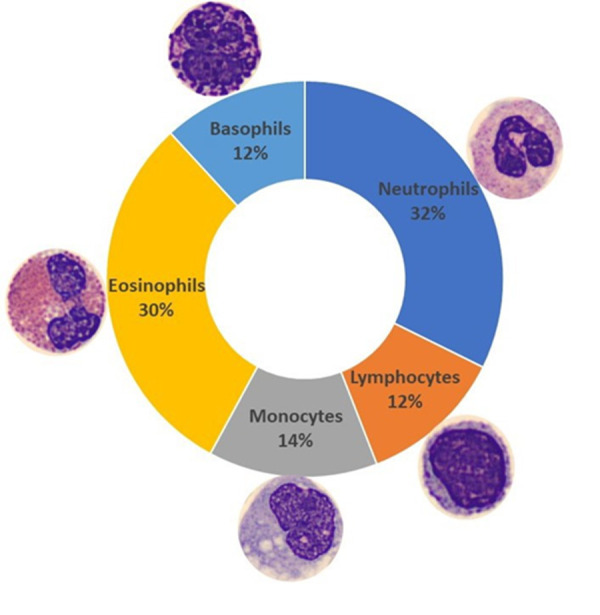
Structure of the dataset used for training the AI algorithm. The percentage of each cell type in the dataset is presented in parenthesis

To create a model for classification of peripheral white blood cells, we used images derived from CellaVision and published previously^8^. Google-based no-code Teachable Machine was used as an AI platform to classify the cells. The total number of cells used for this project was 10298 categorized in 5 classes: neutrophils, lymphocytes, monocytes, eosinophils, and basophils (Figure 1 and Table 1).

**Table 1:** Distribution of specimens by class for training and test sets

Cell type	Total samples	Training samples	Test samples
Neutrophils	3329	2829	500
Lymphocytes	1214	1031	183
Monocytes	1420	1207	213
Eosinophils	3117	2649	468
Basophils	1218	1035	183

**Figure 2:**
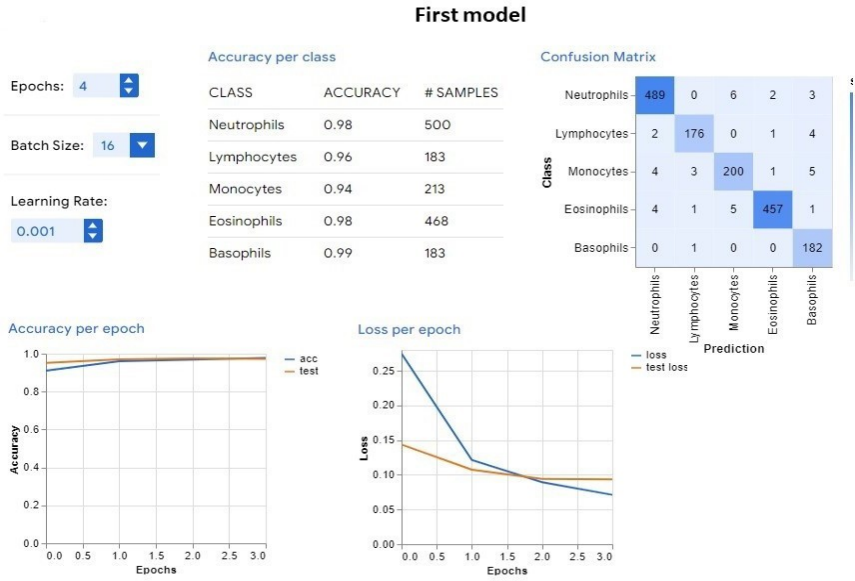
Confusion matrix, accuracy and loss function for the first AI model. The system hyperparameters is shown on the top left. The accuracy per class and the confusion matrix show the performance of the AI model on the test set. The rows of the matrix represent the actual classes, and the columns represent the predicted classes. The diagonal elements of the matrix show the number of instances that were correctly classified, and the off-diagonal elements show the number of instances that were misclassified. The accuracy per epoch shows the accuracy of the AI model on the test set as a function of the number of epochs. The loss per epoch shows the loss of the AI model on the test set as a function of the number of epochs

Teachable Machine devides the cells into 85% for training and 15% for testing the algorithm. This platform allows adjustment of three so-called hyperparameters that can change the learning process of the system: epoch, batch size, and learning rate^12^. An epoch refers to a complete iteration or cycle through the entire dataset during the training phase of a machine learning model. It signifies that the model has processed and learned from all the available training data once. The default epoch number of the Teachable machine is 50. Although the accuracy of the model for the trainning data increased as epoch increases, the accuracy of the model for the test data quickly reached a plateu and did not increase further (Suppl.Figure 1). We tried an epoch of 4 which resulted in comparable or better accuracy compared to an epoch of 50 (Suppl.Figure 2) and it also reduced the time to develop the model. In addition, a higher number of epochs in machine learning can potentially lead to overfitting, where the model becomes too specific to the training data and performs poorly on unseen data^13^. Overfitting occurs when the model learns not only the general patterns in the data but also the noise or random variations present in the training set (memorization). This is reflected in the loss function, which measures the error or discrepancy between the predicted outputs of the model and the actual target values in the training data. As the number of epochs increased (until an epoch of 4), the accuracy of the algorithm improved, and the loss function decreased, indicating a declining error in the system (Figure 2 and Suppl.Figure 1). However, a further increase in the number of epochs led to an increase in loss function (Suppl.Figure 1). Consequently, the model may fail to generalize well to new examples. Therefore we adopted an epoch of 4.

**Figure 3:**
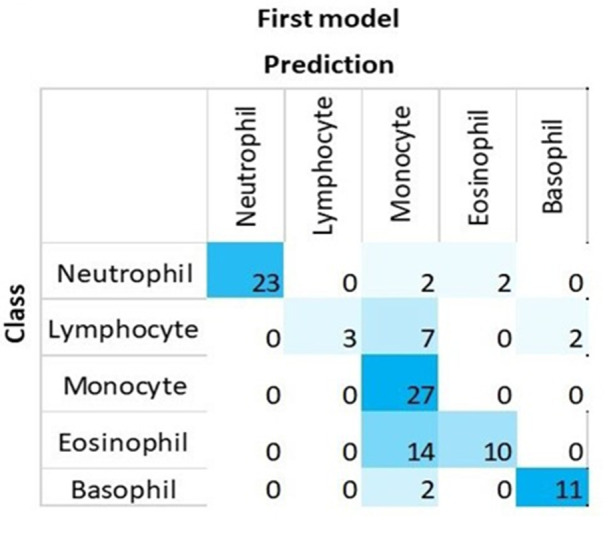
Confusion matrix for the independent images tested on the AI model. The confusion matrix shows the performance of the AI model on a test set of 101 images. The images were sourced from independent sources and consisted of 27 neutrophils, 12 lymphocytes, 27 monocytes, 24 eosinophils, and 13 basophils. The diagonal elements of the matrix show the number of instances that were correctly classified, and the off-diagonal elements show the number of instances that were misclassified

**Figure 4:**
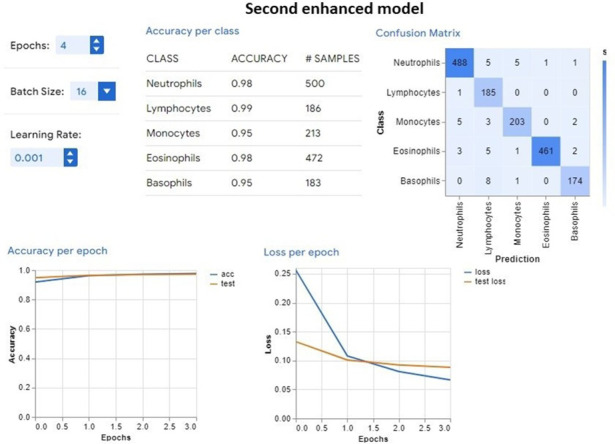
Confusion matrix, accuracy and loss function for the enhanced second AI model. The system hyperparameters is identical to those in ​, but 10 more lymphocytes and 24 more eosinophils from different sources were added to the training dataset to increase cell diversity

The batch size refers to the number of training examples utilized in a single forward and backward pass during the training process^14^. Instead of processing the entire training dataset at once, the data is divided into smaller batches, and the model updates its parameters based on the gradients computed from each batch. We tested several batch sizes (16, 32, and 64) and observed that a higher batch size for our dataset does not improve the accuracy of the model and therefore chose the Teachable Machine default batch size of 16 (Suppl.Figures 2-4).

**Figure 5:**
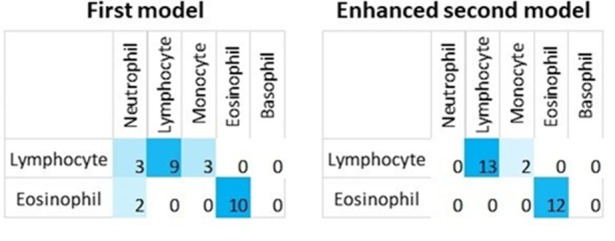
Comparison between the first and the second AI models for classification of lymphocytes and eosinophils

The learning rate determines the step size or rate at which a model's parameters (weights and biases) are updated during the training process^15^. We tested several learning rates (0.001, 0.0005, and 0.0001) and observed that a lower learning rate (0.0005 and 0.0001) does not improve the overal accuracy of the model (Suppl.Figure 2 and Suppl.Figures 5-6). Hence, we chose the default learning rate of 0.001. The lack of significant improvement of the model by tweaking these hyperparameters may be due to the originally

**Table 2:** Performance metrics of the white blood cell classification AI model

Cell type	Sensitivity (Recall)	Precision (Positive predictive value)	F1 score	# Samples
Neutrophils	0.98	0.98	0.98	500
Lymphocytes	0.96	0.97	0.97	183
Monocytes	0.94	0.95	0.94	213
Eosinophils	0.98	0.99	0.98	468
Basophils	0.99	0.93	0.96	183

high classification accuracy that maxed out the outcomes. Although investigating the effect of hyperparameters on AI algorithms is not the focus of our study, using suboptimal parameters with intentionally lower accuracies could allow assessment of these parameter’s effect on the algorithms. The combination of an epoch of 4, batch size of 76, and learning rate of 0.001 provided a model with high accuracy, sensitivity (recall), and precision (positive predictive value) in classifying the cells (Figure 2 and Table 2). The F1 score is a measure that combines two fundamental performance metrics, precision and recall, into a single value providing a balanced measure of a model's effectiveness. 

**Table 3:** Performance metrics of the first AI classifier on independent images (test images from reference ^10^)

Cell type	Sensitivity (Recall)	Precision (Positive predictive value)	F1 score	# Samples
Neutrophils	0.85	1	0.92	27
Lymphocytes	0.25	1	0.4	12
Monocytes	1	0.52	0.68	27
Eosinophils	0.42	0.83	0.56	24
Basophils	0.85	0.85	0.85	13

The models performed well for all five cell types (F1 score >96%). The performance was slightly lower for monocytes (F1 score of 0.94). Since machine learning algorithms learn from the input information, it is crucial to validate the models using independent cohorts. For this purpose, CellaVision-derived images from various sources, were selected. No cell selection was performed to ensure an unbiased assessment of the model. The results showed a sensitivity of approximately 100% for monocytes and 85% for neutrophils and basophils. However, the sensitivity for eosinophils and lymphocytes were 0.42 and 0.25, respectively. (Figure 3 and Table 3). The overal accuracy of the model to classify these new cells was 73%.

One aspect about AI algorithms is that they are dependent on the type of data they are trained with and the diversity of the training datasets help the model to perform well with new data. Because the sensitivity of our AI model for lymphocyte and eosinophil classification was low, the model was trained with additional images. For lymphocytes, the high morphologic diversity necessitates the model to see more representative examples. Therefore, we added 10 more lymphocyte images to the training dataset^9^. For eosinophils, our training dataset contained images with bright red eosinophilic granules but the independent test dataset had eosinophils with dimmer red granules. We hypothesize that this may account for poor sensitivity (0.42). Hence, 24 eosinophil pictures that contained dimmer granules^10^ were added to the images and the model was trained with identical previous hyperparameters (Figure 4). This new enhanced model showed similar high overal accuracy of 97%. To test the effect of new training dataset images on the AI classification sensitivity, the first and the enhanced second model were tested on new independent images^11,16,17^. As shown in Figure 5 and Table 4, the enhanced second model when compared to the first model, has higher sensitivity for classifying lymphocytes (0.6 vs 0.8) and eosinophils (0.83 vs 1), respectively, reiterating the importance of a diverse dataset for training AI algorithms.

**Table 4:** Sensitivity of the first and second AI classifier on independent images (test images from references ^11,16,17^

Cell type	First model	Second model	# Samples
Lymphocytes	0.6	0.8	15
Eosinophils	0.83	1	12

## DISCUSSION

Our study introduces an innovative approach to classifying white blood cells, utilizing a no-code AI model that can be employed by clinicians without coding expertise. We employed a large dataset comprising images from freely available published databases. The performance of our model in white blood cell classification demonstrated high sensitivity and precision. Furthermore, the independent validation of the model using unseen data allowed identifying the role of training database diversity on the AI performance. Importantly, addition of only 10 new lymphocytes to a pool of already 1031 lymphocytes and 24 new eosinophils with slightly dimmer granule color intensity to a pool of 2649 eosinophils (less than 1% increase in the training cell pool) greatly improved the sensitivity of the algorithms when encountered unseen data. To the best of our knowledge, this study represents the first use of a freely available no-code AI platforms in the field of hematopathology and is among the few published studies exploring the application of a no- Code platforms in Medicine^18,19^.

Notably, our research excels in utilizing one of the largest number of distinct white blood cells to train an AI model, resulting in highly accurate and precise classification compared to other models^20^. While some methods exist to expand sample sizes by generating synthetic variants through techniques like image rotation, contrast alteration, noise induction, or stretching, these variations are considered semi- artificial, and although there is some merit in their use, they lack authenticity as biological variants^21^. Moreover, these synthetic variants, being highly similar to the original samples, may artificially inflate the performance metrics of the system. In contrast, our dataset remained unaltered and comprised over 10,000 unique cells, providing a more representative foundation for clinical applications.

A few important caveats in this study: we used images generated by CellaVision machine. Images made by other scanners or taken by a normal camera were not included in the training. This is because our primary goal was to familiarize clinicians and pathologists with AI platforms and allow them to have hands-on experience in developing an AI model with no coding experience and not to generate an all- inclusive AI platform for clinical applications. This preliminary and initial study is for demonstration of the model, and not for identifying abnormal cell types. Additionally, there are various subtypes within each cell type that hold significant clinical relevance. For instance, neutrophils may contain different types of intracellular granules, or the number of nuclear segments may differ, which are associated with specific diseases (e.g., bi-lobed nuclei in Pegler Huet anomaly and myelodysplastic syndrome, presence of intracellular microorganisms in sepsis and anaplasma). The complexity of phenotyping these cells necessitates expert labeling by experienced technologists or hematopathologists, along with correlation with other diagnostic findings such as microbiological tests and flow cytometry. The lack of a large publicly accessible database encompassing immature cells or cells with specific phenotypes for training AI models creates a distinctive research niche.

The utilization of no-code AI models presents a unique opportunity for clinicians, radiologists, pathologists, and other healthcare professionals who have access to high-quality and expert-labeled medical information. It allows them to generate clinically relevant and valuable AI models in their respective fields of study, aiming to enhance patient care. In addition to image-based models, no-code platforms such as Teachable Machine enable the creation of models based on audio and postural (pose) inputs. This opens up exciting possibilities for employing audio inputs in cardiology, pulmonology, vascular surgery, prenatology, and speech pathology. The authors have experience and studied other platforms in histopathology; however, many of these domains are not freely available.

In summary, the recent availability and evolution of no-code AI platforms present an unparalleled opportunity for medical experts to advance their unique ideas and create valuable models that may finally contribute to better patient care.
